# Editorial: Decoding tumor plasticity: integrative analysis of epigenetic regulation and microenvironmental adaptation

**DOI:** 10.3389/fimmu.2026.1783659

**Published:** 2026-01-16

**Authors:** Ran Wang, Haitao Wang, Jian-Guo Zhou, Guan-Jun Yang

**Affiliations:** 1 State Key Laboratory for Managing Biotic and Chemical Threats to the Quality and Safety of Agro-products, School of Marine Sciences, Ningbo University, Ningbo, Zhejiang, China; 2 Center for Cancer Research, National Cancer Institute (NCI), National Institutes of Health (NIH), Bethesda, MD, United States; 3 Department of Oncology, The Second Affiliated Hospital of Zunyi Medical University, Zunyi, China

**Keywords:** cancer stem cells, epigenetic regulation, therapeutic resistance, transdifferentiation, tumor microenvironment, tumor plasticity

Cancer remains one of the most significant challenges in modern medicine, primarily due to its extraordinary capacity for adaptation. Central to this resilience is tumor plasticity—the ability of cancer cells to dynamically shift their phenotypic states in response to intrinsic and extrinsic environmental cues (1). This adaptive behavior underpins key hallmarks of tumor biology, including heterogeneity, therapeutic resistance, metastasis, and disease recurrence, and represents a major barrier to achieving durable clinical responses (2).

Recent advances in cancer biology have challenged the classical view of tumor differentiation as a rigid, unidirectional process toward dedifferentiation. Emerging evidence supports a more dynamic and continuous model, wherein cancer cells occupy a spectrum of phenotypic states, governed by intricate interactions between epigenetic reprogramming and microenvironmental signals. These findings necessitate a paradigm shift in how we conceptualize tumor progression and design therapeutic interventions.

This Research Topic, titled *“Decoding tumor plasticity: Integrative analysis of epigenetic regulation and microenvironmental adaptation”*, brings together cutting-edge contributions that advance our comprehending of the molecular and cellular mechanisms underlying tumor plasticity. The Research Topic includes 12 high-quality articles authored by 79 researchers across diverse disciplines. Collectively, these studies underscore the critical roles of epigenetic regulation and microenvironmental adaptation in shaping tumor cell identity and behavior:

The roles of immune cells in tumor plasticity are complex and dynamic, shaped by continuous interactions between immune cell subsets and the tumor microenvironment (TME). This plasticity enables immune cells to adapt functionally in response to changing TME conditions, thereby modulating tumor progression and therapeutic responses. Natural killer (NK) cells, for instance, play a central role in tumor immunity, exhibiting functional and phenotypic plasticity that can yield either pro- or anti-tumoral outcomes depending on environmental cues (3). Harnessing this adaptability offers promising avenues for developing novel immunotherapeutic strategies. Dong et al. highlighted the critical interaction between NK cells and head and neck squamous cell carcinoma (HNSCC), wherein NK cells exert direct cytotoxic and immunomodulatory effects. However, HNSCC evades NK cell surveillance through mechanisms such as downregulation of NKG2D ligands and the establishment of an immunosuppressive TME. Cetuximab, an anti-EGFR monoclonal antibody, enhances NK cell-mediated antibody-dependent cellular cytotoxicity and immunogenic modulation, improving outcomes in EGFR-overexpressing HNSCC. Emerging approaches—including CAR-NK cells, cytokine-primed NK cells, and bispecific engagers—show clinical potential. However, challenges such as TGF-β-mediated suppression and metabolic competition within the TME must be addressed to optimize NK cell-based therapies. Combining EGFR inhibition with NK cell activation may offer a synergistic strategy for HNSCC treatment. In colorectal cancer (CRC), impaired NK cell infiltration and function within the TME have been observed, although the underlying mechanisms remain incompletely understood. Li et al. reported reduced SMAD4 expression in tumor-infiltrating NK cells, with SMAD4 overexpression enhancing NK cell cytotoxicity against CRC cells. Mechanistically, SMAD4 upregulates NKG2D expression via YTHDF2, a key downstream effector that regulates m6A-modified RNA metabolism. These findings establish the SMAD4–YTHDF2 axis as a critical regulator of NK cell anti-tumor activity and suggest SMAD4 as a potential therapeutic target for enhancing NK cell-based immunotherapy in CRC.

Similarly, tumor-associated macrophages (TAMs) exhibit plasticity that allows them to facilitate immune evasion and promote tumor metastasis. TAMs can release cytokines that inhibit effector immune cells and attract additional immunosuppressive cells to the TME, highlighting their role as key regulators of tumor immune evasion (4). Lung cancer (LC) remains a leading global malignancy, where tumor-associated macrophages (TAMs) is implicated in tumor progression and metastasis through their dynamic polarization into pro-tumor (M2-like) or anti-tumor (M1-like) phenotypes. The plasticity of TAMs, governed by complex signaling crosstalk within the TME, offers therapeutic opportunities to reprogram M2-like macrophages toward tumor-suppressive M1-like states. Li et al’s works highlight synthetic and natural compounds that modulate these pathways, while nanotechnology-enabled drug delivery systems (NDDSs) enhance precision and efficacy by targeting TAMs with minimal off-target effects. This review systematically examines the signaling networks between TAMs and LC cells, surveys cutting-edge TAM-reprogramming therapeutics, and evaluates nano-based strategies for combination therapy. Despite promising preclinical progress, we also address the challenges and future directions for translating TAM-targeted therapies into clinical practice. Liu et al.’s study demonstrated that SHP2^+^ TAMs serve as a theranostical target in non-small cell lung cancer (NSCLC). Through multiplex fluorescence analysis of 79 NSCLC specimens, we revealed that CD68^+^SHP2^+^ TAMs are predominantly enriched in tumor regions (vs stroma) and correlate with poor overall survival (p < 0.05). Importantly, SHP2 expression was higher in M2-like (CD68^+^CD206^+^) than M1-like (CD68^+^CD86^+^) macrophages, with strong positive correlations between CD68^+^SHP2^+^ and CD68^+^CD206^+^ subsets across all TME compartments (p < 0.0001). These findings position SHP2 as a key regulator of M2-like TAM polarization and suggest that SHP2 inhibition could reprogram the immunosuppressive TME, offering a novel immunotherapeutic strategy for NSCLC. The spatial distribution patterns of SHP2^+^ TAM subpopulations provide critical insights for developing targeted therapies and predictive biomarkers.

The occurrence and development of tumor plasticity is synergistically driven by the interaction between intrinsic regulation and the external microenvironment, in which the intrinsic regulatory network of tumor cells themselves plays a core leading role, and its importance is comparable to the interaction effect between the tumor microenvironment and immune cells (5). Moreover, the key molecules in the regulatory network can govern the formation of malignant phenotypes and the establishment of environmental adaptability of tumor cells by regulating biological processes such as metabolism, epigenetics and cell cycle, and they also serve as core targets for the development of precision targeted therapy strategies (6). Dysregulated lipid metabolism is a key hallmark of prostate adenocarcinoma progression (7). Wang et al. study elucidates the critical role of stearoyl-CoA desaturase (SCD) in prostate adenocarcinoma (PRAD) progression through comprehensive multi-omics analyses. We identified elevated SCD expression in PRAD, associated with promoter hypermethylation and the L134V mutation, which correlated with increased tumor aggressiveness. Intriguingly, SCD levels showed positive associations with CD8+ T cell and macrophage infiltration, suggesting immunometabolic crosstalk in the tumor microenvironment. Single-cell RNA sequencing revealed cell-type specific SCD expression patterns, while functional enrichment analyses linked SCD to lipid/cholesterol biosynthesis and cell cycle pathways. Crucially, experimental validation demonstrated that SCD overexpression enhanced PRAD cell proliferation and invasion, while its knockdown exerted opposite effects. These findings position SCD as both a promising therapeutic target and prognostic biomarker in PRAD, particularly given the tumor’s unique dependence on lipid metabolism. Tumor plasticity and immune microenvironment remodeling are the core drivers of gastric cancer malignant progression. Ye et al.’s study reveals the oncogenic role of AMBRA1 in driving stomach adenocarcinoma (STAD) progression through modulation of tumor plasticity and immune interactions. Comprehensive analyses demonstrated that elevated AMBRA1 expression correlates with poor patient survival and is associated with CD4+ T cell infiltration and cancer-associated fibroblast activity. Functional investigations showed that AMBRA1 depletion impairs STAD cell proliferation, migration, and invasion by inducing G1/S cell cycle arrest and promoting cellular senescence through H3K9me3-mediated epigenetic regulation. Notably, AMBRA1 inhibition enhanced chemosensitivity and significantly attenuated tumor growth in xenograft models. These findings position AMBRA1 as a promising therapeutic target, with dual mechanisms of action involving both tumor-intrinsic senescence induction and potential immune microenvironment modulation, offering new avenues for precision therapy in STAD. Dysregulated ribosome biogenesis and immune evasion are crucial mechanisms underlying the initiation and progression of B-cell malignancies (8). Li et al. identified ribosomal protein L9 (RPL9) as a key oncogenic driver in B-cell acute lymphoblastic leukemia (B-ALL), operating through dual mechanisms. RPL9 is significantly overexpressed in B-ALL cells relative to normal B lymphocytes, and its knockdown (KD) inhibits proliferation, induces apoptosis *in vitro*, and prolongs survival in B-ALL xenografted NCG mice. Mechanistically, RPL9 KD disrupts ribosome biogenesis, triggers nucleolar stress, and activates the p53 pathway—an effect reversible by FTO overexpression, consistent with prior findings linking FTO-mediated m6A modification to RPL9 regulation. Notably, RPL9 depletion also enhances susceptibility to NK cell-mediated cytotoxicity by upregulating MICA/B, ligands for the NKG2D receptor. These findings establish RPL9 as a promising dual-function therapeutic target, offering a synergistic strategy that combines tumor suppression via p53 activation with immunotherapy enhancement through NK cell engagement.

Childhood tumors, as malignant diseases that seriously threaten the life and health of adolescents, have long been limited by the predicaments of strong toxicity, high invasiveness and poor prognosis associated with traditional radiotherapy and chemotherapy, and the treatment response rate and quality of life of patients with high-risk cases still need to be urgently improved (9, 10). Neuroblastoma (NB), the most common pediatric extracranial solid tumor, remains challenging to treat, particularly in high-risk cases, with 5-year survival rates below 50% despite GD2-targeting immunotherapies. Characterized as immunologically “cold,” NB tumors evade immune detection through impaired antigen presentation and immunosuppressive TMEs. The dysregulation of epigenetic mechanisms, such as DNA methylation, histone modifications, and chromatin remodeling, contributes to NB pathogenesis and justifies the pursuit of targeted therapies. Wang et al. reviewed the therapeutic potential of histone deacetylase inhibitors and DNA methyltransferase inhibitors, which offer advantages in development speed and cost-effectiveness. These agents may overcome the limitations of current immunotherapies and improve outcomes for high-risk NB patients.

Retinoblastoma, the most common pediatric ocular malignancy, presents significant therapeutic challenges due to the severe complications—such as vision loss and systemic toxicity—associated with conventional treatments like chemotherapy, radiotherapy, and surgery. GD2-targeted immunotherapy offers a promising alternative by exploiting the tumor-specific expression of disialoganglioside GD2 while sparing normal tissues. Zhang et al. summarized recent advances in GD2-directed therapies, including: (1) Dinutuximab, an FDA-approved anti-GD2 monoclonal antibody initially developed for neuroblastoma and now under evaluation in retinoblastoma; (2) GD2-targeted CAR-T cell therapy; (3) GD2-based vaccines to stimulate adaptive immunity; and (4) nanoparticle delivery systems for enhanced targeting. These approaches demonstrate improved safety profiles and better quality-of-life outcomes compared to traditional modalities. However, challenges remain, particularly in achieving effective intraocular drug penetration, managing immune-related adverse events, and countering tumor immune evasion. This review critically assesses the current preclinical and clinical landscape of GD2 immunotherapy and proposes strategies—such as combination therapies and novel delivery systems—to overcome existing limitations.

The individualized and precise regulation of immunotherapy is the key to improving diseases and their prognosis, and dynamic monitoring strategies during treatment are important supports for achieving this goal. The heterogeneity of B-cell regeneration kinetics poses challenges to treatment guidance (11). Therefore, exploring the clinical application value of B-cell monitoring is of great practical significance. Dong et al. evaluated the clinical utility of B cell monitoring in 488 patients with autoimmune and transplant-related diseases undergoing rituximab (RTX) therapy from 2017 to 2024. While most patients exhibited B cell repopulation by 24 weeks post-treatment, a subset (6.8%) showed early repopulation within 4 weeks. Significant interpatient variability in B cell recovery kinetics was observed, underscoring the importance of regular B cell monitoring to guide individualized immunotherapy decisions, optimize treatment timing, and predict disease remission or recurrence. These findings support the implementation of standardized B cell surveillance protocols across diverse clinical indications.

Epstein-Barr virus (EBV) infection is widespread and closely associated with a variety of malignant tumors and autoimmune diseases. Traditional EBV vaccine development is limited by bottlenecks such as insufficient antigen immunogenicity and poor targeting, while the combination of nanoparticle delivery systems and precise antigen epitope screening provides a new approach to break through this predicament. Li et al.’s developed a novel nanoparticle-based vaccine targeting Epstein-Barr virus (EBV), addressing the urgent need for effective EBV prophylaxis. The vaccine features a glycan-free recombinant protein (L350), comprising five epitopes from the EBV gp350 glycoprotein’s receptor-binding domain, validated for binding to host AKATA cells. L350 was displayed on self-assembling ferritin nanoparticles to enhance immunogenicity. In Balb/c mice, the L350-ferritin vaccine elicited strong antibody responses against both L350 and native gp350, and established durable antigen-specific B-cell memory. Importantly, vaccinated sera exhibited potent neutralizing activity, effectively inhibiting EBV-GFP infection *in vitro*. No histopathological abnormalities were observed, underscoring the vaccine’s safety. These results position L350-ferritin as a promising EBV vaccine candidate, integrating rational epitope design with nanoparticle delivery to overcome prior development hurdles. The study provides both a specific prophylactic candidate and a versatile platform for future EBV vaccine development.

This synthesis highlights how understanding immune cell plasticity in the TME informs the development of targeted therapies across diverse malignancies, while underscoring the need for continued investigation into combination approaches and treatment optimization strategies.
